# [Corrigendum] Corneal endothelial changes induced by pars plana vitrectomy with silicone oil tamponade for retinal detachment

**DOI:** 10.3892/etm.2022.11747

**Published:** 2022-12-06

**Authors:** Corina Cristina Coman (Cernat), Mihnea Munteanu, Daniel Malita, Simona Stanca, Stella Ioana Patoni (Popescu), Ovidiu Musat, Serban Negru, Horea Feier, Olimpiu Ladislau Karancsi, Cosmin Rosca

Exp Ther Med 22:961, 2021; DOI: 10.3892/etm.2021.10393

Subsequently to the publication of the above article, the first author [Corina Cristina Coman (Cernat)] contacted the Editorial office to explain that the name of her PhD coordinator (Dr Mihnea Munteanu) was inadvertently omitted from the paper. Dr Munteanu should have been listed as the second author on the paper; therefore, the revised author list and the affiliations for the authors should have appeared as follows:

CORINA CRISTINA COMAN (CERNAT)^1*^, MIHNEA MUNTEANU^1*^, DANIEL MALITA^2*^, SIMONA STANCA^3*^, STELLA IOANA PATONI (POPESCU)^1^, OVIDIU MUSAT^4^, SERBAN NEGRU^5^, HOREA FEIER^6^, OLIMPIU LADISLAU KARANCSI^7^ and COSMIN ROSCA^8^

Departments of ^1^Ophthalmology, and ^2^Radiology and Medical Imaging, ‘Victor Babes’ University of Medicine and Pharmacy, 300041 Timisoara; ^3^Department of Pediatrics, ‘Carol Davila’ University of Medicine and Pharmacy, 050474 Bucharest; ^4^Department of Ophthalmology, ‘Dr. Carol Davila’ Central Military Emergency University Hospital, 010825 Bucharest; ^5^Department of Oncology, ‘Victor Babes’ University of Medicine and Pharmacy, 300239 Timisoara; Departments of ^6^Cardiovascular Surgery, and ^7^Oral Implantology and Prosthetic Restorations on Implants, ‘Victor Babes’ University of Medicine and Pharmacy, 300041 Timisoara; ^8^Department of Ophthalmology, Oculens Clinic, 400501 Cluj-Napoca, Romania

^*^Contributed equally

The authors regret that Dr Munteanu’s name was not originally included on the paper, although all the authors agree to this change in the authorship. Furthermore, the authors apologize to the readership for any inconvenience caused.

